# Evidence for the complex relationship between free amino acid and sugar concentrations and acrylamide-forming potential in potato

**DOI:** 10.1111/aab.12101

**Published:** 2014-01-23

**Authors:** N Muttucumaru, SJ Powers, JS Elmore, A Briddon, DS Mottram, NG Halford

**Affiliations:** 1 Plant Biology and Crop Science Department, Rothamsted ResearchHarpenden, UK; 2 Computational and Systems Biology Department, Rothamsted ResearchHarpenden, UK; 3 Department of Food and Nutritional Sciences, University of ReadingReading, UK; 4 Potato Council, Sutton Bridge Crop Storage ResearchSpalding, UK

**Keywords:** Acrylamide, amino acids, food safety, post-harvest storage, potato, process contaminant, sugars

## Abstract

Free amino acids and reducing sugars participate in the Maillard reaction during high-temperature cooking and processing. This results not only in the formation of colour, aroma and flavour compounds, but also undesirable contaminants, including acrylamide, which forms when the amino acid that participates in the reaction is asparagine. In this study, tubers of 13 varieties of potato (*Solanum tuberosum*), which had been produced in a field trial in 2010 and sampled immediately after harvest or after storage for 6 months, were analysed to show the relationship between the concentrations of free asparagine, other free amino acids, sugars and acrylamide-forming potential. The varieties comprised five that are normally used for crisping, seven that are used for French fry production and one that is used for boiling. Acrylamide formation was measured in heated flour, and correlated with glucose and fructose concentration. In French fry varieties, which contain higher concentrations of sugars, acrylamide formation also correlated with free asparagine concentration, demonstrating the complex relationship between precursor concentration and acrylamide-forming potential in potato. Storage of the potatoes for 6 months at 9°C had a significant, variety-dependent impact on sugar and amino acid concentrations and acrylamide-forming potential.

## Introduction

Reducing sugars, such as glucose and fructose, react with free amino acids during high-temperature cooking and processing (frying, baking and roasting, but not boiling) in a series of non-enzymatic reactions given the umbrella name of the Maillard reaction (Halford *et al.*, 2011; Mottram, 2007; Nursten, 2005). The Maillard reaction results in the formation of a plethora of products, many of which impart colour, aroma and flavour. However, it also gives rise to some undesirable contaminants, including acrylamide. Acrylamide forms when the amino acid that participates in the reaction is asparagine (Mottram *et al.*, 2002; Stadler *et al.*, 2002; Zyzak *et al.*, 2003). For simplicity we will refer to free asparagine and reducing sugars as precursors of acrylamide, although the carbon skeleton of acrylamide is derived entirely from asparagine. It should also be noted that other routes for acrylamide formation have been proposed (Claus *et al.*, 2006; Granvogl *et al.*, 2004).

Acrylamide has been shown to be carcinogenic and to have neurological and reproductive effects in rodent toxicology studies (Friedman, 2003). As a result, it has been classified as a Group 2A, ‘probably carcinogenic to humans’, chemical by the International Agency for Research on Cancer. The Food and Agriculture Organisation of the United Nations and the World Health Organisation (FAO/WHO) Joint Expert Committee on Food Additives (JECFA) recently concluded that the margin of exposure for acrylamide (the ratio of the level at which a small but measurable effect is observed to the estimated exposure dose) indicates that its presence in the human diet is a concern and that epidemiological studies using haemoglobin adducts of acrylamide itself, or its primary epoxide metabolite, glycidamide, as a measure of exposure were required to estimate the risk (Joint FAO/WHO Expert Committee on Food Additives, 2011). To date, however, the results of epidemiological studies have been inconsistent. A recent meta-analysis of epidemiological data, for example, led the authors to conclude that there was no relationship between dietary acrylamide intake and cancer (Lipworth *et al.*, 2012), while a later Danish study did find a link between acrylamide exposure and breast cancer-specific mortality (Olsen *et al.*, 2012). Another recent study showed a link between haemoglobin adducts of acrylamide and glycidamide in umbilical cord blood (reflecting exposure in the last months of pregnancy) and low birth weight and head circumference in babies (Pedersen *et al.*, 2012).

Given this uncertainty regarding the human health risk from acrylamide in the diet and the low margin of exposure, JECFA and other risk assessment bodies have recommended that acrylamide levels in food be reduced as a matter of priority and the European Commission issued ‘indicative’ levels for acrylamide in food in early 2011 (European Commission, 2011). Indicative values are not safety thresholds, but are intended to indicate the need for an investigation into why the level has been exceeded. Fried potatoes (such as French fries) and potato crisps are important contributors to dietary intake across Europe (European Food Safety Authority, 2011) and indicative levels have been set at 1000 µg kg^−1^ (parts per billion; ppb) for crisps and 600 µg kg^−1^ for French fries. The food industry has devised many strategies for reducing acrylamide formation by modifying food processing (compiled in a ‘Toolbox’ produced by Food Drink Europe: http://www.fooddrinkeurope.eu/uploads/publications_documents/Toolboxfinal260911.pdf), and in Europe this has resulted in a significant downward trend for mean levels of acrylamide in potato crisps from 763 µg kg^−1^ in 2002 to 358 µg kg^−1^ in 2011, a decrease of 53% (Powers *et al.*, 2013). Nevertheless, processors remain vulnerable to fluctuations in the acrylamide-forming potential of the crop products that make up their raw material. Developing best practice for crop cultivation and management, alongside variety selection and improvement, therefore, has an important part to play in acrylamide reduction strategies (Muttucumaru *et al.*, 2008; Halford *et al.*, *a*^2012^).

A major management factor affecting the composition of potatoes is post-harvest storage. Glucose and fructose, for example, accumulate rapidly in stored potato tubers in response to the temperature falling below approximately 8°C (cold sweetening; Sowokinos, 1990), sprouting (dormancy break) or tuber senescence after long-term storage. Tubers are therefore usually stored at 8–10°C to prevent cold sweetening, while sprouting, which would occur at this temperature, is controlled by spraying with sprout suppressants such as chlorpropham (CIPC).

The relationship between the concentrations of reducing sugars (principally glucose and fructose, with very little maltose), free asparagine and other amino acids in potato tubers and acrylamide formation during cooking and processing is complicated, with separate studies concluding reducing sugar concentration, free asparagine concentration or free asparagine concentration as a proportion of the total free amino acid pool to be the determining factor (Amrein *et al.*, 2003; Becalski *et al.*, 2004; Elmore *et al.*, 2007, 2010; Shepherd *et al.*, 2010). Recently, clear correlations have been shown between reducing sugar concentration and acrylamide-forming potential in nine varieties of potatoes grown commercially in the UK in 2009 (Halford *et al.*, *b*^2012^), but free asparagine and total free amino acid concentration also correlated significantly with acrylamide-forming potential in French fry but not crisping (US chipping) varieties, probably because French fry varieties contain higher concentrations of sugars. Another recent study modelled the kinetics of acrylamide formation in French fry production and concluded that both the fructose/glucose ratio and the ratio of asparagine to total free amino acids could affect acrylamide formation (Parker *et al.*, 2012).

Clearly, the relationship between precursor concentration and acrylamide formation must be understood to enable food producers to select the most suitable varieties as the raw material for their products and to develop quality control measures where appropriate. A comprehensive understanding of the relationship is also essential for the identification of target traits for breeders. The aim of this study, therefore, was to add to the data on this complex issue by analysing 13 varieties of potatoes that had been grown in a field trial in 2010 (Muttucumaru *et al.*, 2013). The study also investigated the effect of storage on precursor concentration and acrylamide-forming potential.

## Materials and methods

### Potatoes

The potatoes comprised 13 varieties that had been produced in a field trial conducted at the Rothamsted farm site at Woburn, UK, in 2010 (Muttucumaru *et al.*, 2013). Nitrogen (N) had been applied as ammonium nitrate at 0, 100 and 200 kg ha^−1^. Different levels of sulphur (S) had also been applied but all of the potatoes used for this study had been grown with S supplied (as gypsum: CaSO_4_·2H_2_O) at 15 kg S ha^−1^. The potatoes had also received triple super phosphate (CaH_2_PO_4_)_2_·H_2_O) at 128 kg ha^−1^ and muriate of potash (KCl) at 458 kg ha^−1^, and the trial had been irrigated when required. The design of the field trial was a split-plot in three blocks (replicates), with N and S combinations on main plots (for ease of application) and varieties on split-plots, with five plants per plot. Two of the three blocks were taken through to the storage part of the trial.

The tubers had been harvested from 28 September to 28 October 2010; some (the unstored sample) were analysed at harvest, while the rest (the stored sample) were kept in a long-term storage facility at Sutton Bridge Crop Storage Research, UK, at 9°C until April 2011 and were analysed from the 5th to the 7th of that month. The sprout suppressant, CIPC was applied at 14 g per tonne on 12th November, 10th December and 23rd February.

### Free amino acids, sugars and acrylamide formation

Free amino acids and sugars were measured in flour prepared from individual freeze-dried tubers. Free amino acids were derivatised and then analysed by gas chromatography–mass spectrometry (GC–MS) using an Agilent 5975 system (Agilent, Santa Clara, CA, USA) in electron impact mode, as described previously (Halford *et al.*, *b*^2012^). Note that arginine cannot be measured using this system, while cysteine concentrations were too low to measure accurately. Histidine data were acquired for the unstored samples but a problem with the injection port liners supplied with the EZFaast kit resulted in histidine giving variable peaks when the stored samples were being analysed and the data were considered unreliable.

Sugar concentrations were measured using a Dionex ion chromatography system with a 250 × 4 mm Carbopac™ PA1 column (Dionex Corporation, Sunnyvale, CA, USA), operated using Chromeleon™ software, also as described previously (Halford *et al.*, *b*^2012^). Acrylamide was measured in cooked potato flour after heating to 160°C for 20 min. The analysis was performed by liquid chromatography-tandem mass spectrometry (LC–MS/MS) using an Agilent 1200 high performance liquid chromatography (HPLC) system with 6410 triple quadrupole mass spectrometer with electrospray ion source in positive ion mode, as previously described (Halford *et al.*, *b*^2012^).

### Statistical analyses

Analysis of variance (ANOVA) was used for each measured variable to assess the overall significance (*F*-tests) of main effects and interactions between varieties nested within cooking type (French fry, crisping or boiling), N and storage. For this analysis, the design was taken as a split-split plot, the second split reflecting the pairs of samples (unstored and stored) having derived from the same split-plot. Means of interest were then compared using corresponding least significant difference (LSD) values at 5% based on the residual degrees of freedom (df) from the ANOVA. A natural log (to base *e*) transformation was used for all variables to account for some heterogeneity of variance, residuals then conforming to the assumptions of ANOVA. Pearson’s correlation coefficient (*r*) was calculated for variables of interest, for French fry and crisping varieties separately and together. Correlations were tested for statistical significance using the *F*-test. A multiple linear regression model for the transformed acrylamide data was then fitted, using the method of stepwise forward selection of most significant (*P* < 0.05, *F*-test) explanatory variables, then factors and finally interactions between terms already in the model. The GenStat (15th edition, © VSN International Ltd, Hemel Hempstead, UK) statistical package was used for these analyses.

## Results

### Differences in free amino acid and sugar concentrations between types (French fry, crisping and boiling) and varieties of potato, and effects of storage

Thirteen varieties of potato had been grown in a randomised field trial at Woburn in Bedfordshire, UK, in 2010. The varieties comprised seven that are normally used for French fry production (Maris Piper, Pentland Dell, King Edward, Daisy, Markies, Russet Burbank and Umatilla Russet), five that are normally used for crisps (Lady Claire, Lady Rosetta, Saturna, Hermes and Verdi), and one that is generally recommended only for boiling (Harmony), although it is also sometimes described as suitable for baking.

The potato plants had been grown in three replicated blocks, each containing 45 plants of each of the 13 varieties. They had been supplied with different combinations of N and S fertiliser (N at 0, 100 or 200 kg ha^−1^ and S at 0, 15 or 40 kg ha^−1^). This had shown that N application could increase the acrylamide-forming potential in potatoes but that the effect was type- (French fry, crisping and boiling) and variety-dependent, while S application reduced glucose concentrations and mitigated the effect of high N application on the acrylamide-forming potential of some of the French fry-type potatoes (Muttucumaru *et al.*, 2013). This study used two of the replicate blocks and analysed potatoes from plants that had been supplied with N at 0, 100 and 200 kg ha^−1^ together with S at 15 kg ha^−1^ and that were either unstored or following storage. This gave potatoes with a wide range of precursor concentrations while keeping the number of biochemical analyses required at a feasible number. The application of S at 15 kg ha^−1^ was considered to be the closest of the three S treatments to the reality of commercial potato cultivation. Farmers rarely fertilise potatoes with S; however, the soil at the trial site is a sandy loam with very poor nutrient retention, with intrinsic S concentrations ranging from only 0.5–1.8 mg kg^−1^ (Riley *et al.*, 2002).

Free amino acid and sugar concentrations were determined at harvest or shortly after (unstored samples) and after 6 months storage at 9°C in a commercial storage facility. Acrylamide formation was measured in flour after heating at 160°C for 20 min. This method has already been used in several studies because it gives high levels of acrylamide formation, providing a good, consistent indication of acrylamide-forming potential in different raw materials (Curtis *et al.*, 2009, 2010; Elmore *et al.*, 2007; Halford *et al.*, *b*^2012^; Muttucumaru *et al.*, 2006; Postles *et al.*, 2013). Note that free amino acid and acrylamide data were not obtained for unstored Daisy, Umatilla Russet and Harmony because of spoilage of the samples. Note also that arginine cannot be assayed by the method used, while cysteine concentrations were so low that they could not be measured accurately and histidine was omitted from the analysis of the stored samples because of an instrumentation problem. The entire dataset is presented in Appendix S1, Supporting Information.

**Figure 1:**
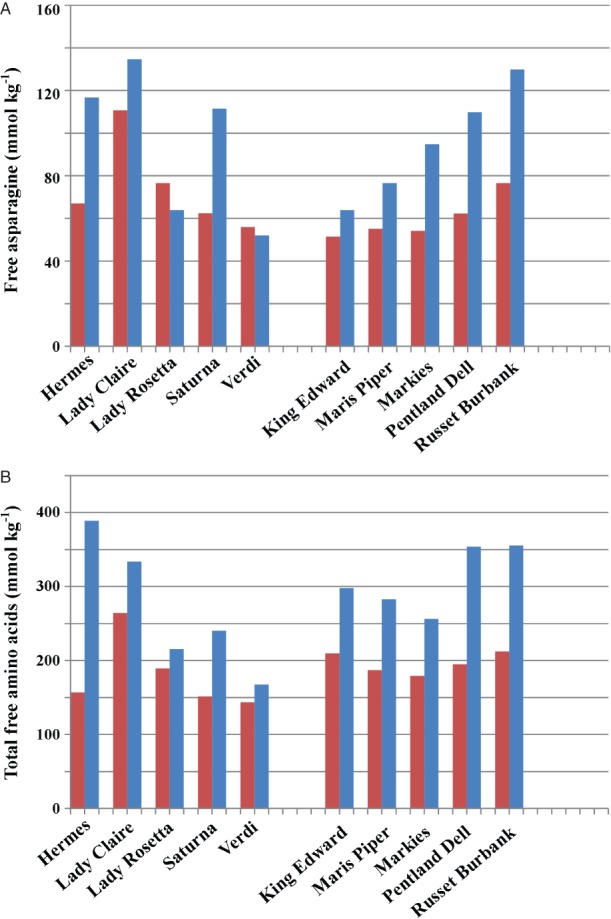
Free asparagine concentrations (A) (mmol kg^−1^ dry weight) and total free amino acid concentrations (B) (mmol kg^−1^ dry weight) (back-transformed means from analysis of variance) in ten potato varieties before storage (red) and after storage (blue). Data were not obtained for the other three varieties in the study (Daisy, Umatilla Russet and Harmony) due to spoilage of the samples. The total free amino acids did not include arginine, cysteine or, for the stored samples, histidine. Statistical analysis of the data is given in Tables 1 and 2.

**Figure 2:**
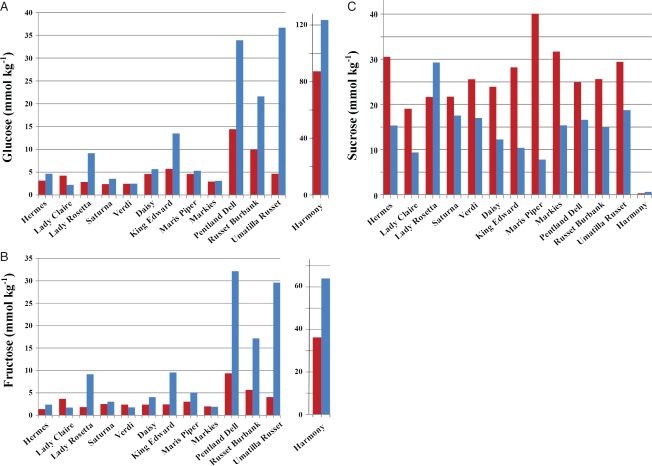
Concentrations of glucose (A), fructose (B) and sucrose (C) (mmol kg^−1^ dry weight) (back-transformed means from analysis of variance) in 13 potato varieties before storage (red) and after storage (blue). Harmony is shown separately on a different scale in (A) and (B) because of its relatively high concentrations of glucose and fructose. Statistical analysis of the data is given in Tables 1 and 2.

The data were subjected to ANOVA, and the resulting *P*-values are given in Table 1. The analysis revealed significant differences between the types and varieties nested within types for free asparagine, many other amino acids, total free amino acids and the sugars with the exception of sucrose, which showed an effect of type but not variety. Type and variety nested within type also showed an interaction with storage, indicating that the types and varieties within types responded differently to storage, consistent with a previous study of commercially-grown potatoes (Halford *et al.*, *b*^2012^). The relevant means on the natural log_e_ scale for free asparagine, total free amino acids and the sugars are given in Table 2 and the back-transformed means are plotted for free asparagine and total free amino acids in Fig. 1 and for the sugars in Fig. 2.

**Table 1:** *P*-Values denoting significance of main effects and interactions of type of potato (T), variety (V) and storage (St), with variety being nested within type, in ANOVA analyses of measured variables on the log_e_ scale for unstored and stored potato samples^a^

	T	T.V	St	T.St	T.V.St
Asparagine	**0.046**	**<0.001**	**<0.001**	0.170	**0.003**
Glutamine	**<0.001**	**<0.001**	0.473	0.333	**<0.001**
Alanine	**<0.001**	**<0.001**	0.088	0.351	0.116
Aspartate	**<0.001**	**<0.001**	**<0.001**	0.932	**0.001**
GABA	**0.004**	**<0.001**	**<0.001**	**0.041**	**<0.001**
Glutamate	0.895	**0.040**	**<0.001**	**0.022**	**0.009**
Glycine	**<0.001**	0.033	0.901	0.357	**0.002**
Isoleucine	**0.009**	**<0.001**	**<0.001**	0.812	0.177
Leucine	0.079	**0.005**	**<0.001**	0.370	0.398
Lysine	0.147	**<0.001**	**<0.001**	0.772	**0.013**
Methionine	**<0.001**	**0.028**	**0.018**	0.396	0.414
Ornithine	0.250	0.168	**0.007**	0.229	0.482
Phenylalanine	**0.006**	**<0.001**	**<0.001**	0.671	0.107
Proline	**<0.001**	**0.002**	**<0.001**	0.649	0.063
Serine	**<0.001**	**0.001**	**<0.001**	0.472	**<0.001**
Threonine	**<0.001**	**<0.001**	**<0.001**	0.269	**0.007**
Tryptophan	0.258	**<0.001**	0.765	0.214	0.296
Tyrosine	0.067	**<0.001**	**<0.001**	0.760	**0.018**
Valine	**<0.001**	**<0.001**	**<0.001**	0.580	**0.042**
Total amino acids	**0.011**	**<0.001**	**<0.001**	0.452	**0.020**
Acylamide	**<0.001**	**<0.001**	**<0.001**	**0.045**	**0.003**
Fructose	**<0.001**	**<0.001**	**<0.001**	**<0.001**	**<0.001**
Glucose	**<0.001**	**<0.001**	**<0.001**	0.068	**<0.001**
Sucrose	**<0.001**	0.854	**<0.001**	**<0.001**	**<0.001**

a Note that arginine and histidine were not assayed, while cysteine concentrations were too low to be measured accurately. A dot indicates interaction between any of the treatment factors. *P*-Values in bold indicate the significant (*P* < 0.05, *F*-test) ANOVA terms. See text in Results section for details of effects involving N.

**Table 2:** Variety nested within type by storage means (*n* = 12) for free asparagine (mmol kg^−1^ dry weight), total free amino acids (mmol kg^−1^ dry weight), glucose, fructose and sucrose (mmol kg^−1^ dry weight) on the natural log_e_ scale, following ANOVA analyses, for stored and unstored potato samples^a^

Type	Variety	Asparagine		Total Amino Acids		Glucose		Fructose		Sucrose	
		Unstored	Stored	Unstored	Stored	Unstored	Stored	Unstored	Stored	Unstored	Stored
**Crisping**	**Hermes**	4.205	5.116	5.055	5.963	1.147	1.544	0.338	0.865	3.418	2.727
	**Lady Claire**	4.707	4.903	5.577	5.810	1.434	0.801	1.293	0.533	2.947	2.237
	**Lady Rosetta**	4.338	4.157	5.243	5.373	1.047	2.214	0.609	2.212	3.074	3.377
	**Saturna**	4.133	4.714	5.020	5.482	0.878	1.259	0.919	1.108	3.077	2.864
	**Verdi**	4.025	3.951	4.966	5.122	0.886	0.920	0.878	0.569	3.242	2.832
**French fry**	**Daisy**	^b^	^b^	^b^	^b^	1.536	1.736	0.862	1.405	3.173	2.507
	**King Edward**	3.941	4.157	5.345	5.697	1.739	2.600	0.889	2.256	3.338	2.341
	**Maris Piper**	4.009	4.338	5.231	5.644	1.522	1.672	1.122	1.628	3.691	2.050
	**Markies**	3.990	4.552	5.189	5.546	1.082	1.135	0.688	0.622	3.457	2.733
	**Pentland Dell**	4.132	4.699	5.272	5.869	2.671	3.523	2.241	3.470	3.217	2.810
	**Russet Burbank**	4.338	4.866	5.359	5.874	2.298	3.073	1.729	2.842	3.242	2.709
	**Umatilla**	^b^	^b^	^b^	^b^	2.322	3.603	1.403	3.386	3.381	2.931
**Boil**	**Harmony**	^b^	^b^	^b^	^b^	4.469	4.817	3.591	4.175	−1.151	−0.463
**SED for means with same variety (29 df)**		0.1727		0.1434		0.2338		0.2389		0.1345	
**LSD (5%)**		0.3531		0.2938		0.4783		0.4885		0.2751	
**SED for other comparisons (df)**		0.1791		0.1537 (53)		0.3490 (52)		0.3166 (57)		0.3587 (40)	
**LSD (5%)**		0.3589		0.3082		0.7001		0.6339		0.7247	

a The standard error of the difference (SED), degrees of freedom (df) and the least significant difference (LSD) values at the 5% level of significance for comparison of means on the log_e_ scale are also included. Note that the total free amino acids data did not include arginine, cysteine or, for the stored samples, histidine.

b Not in analysis because of spoilage of the unstored samples.

There was a trend for free asparagine and total free amino acid concentrations to rise during storage (Fig. 1), with significant (*P* < 0.05, LSD) increases in free asparagine for varieties Hermes, Saturna, Markies, Pentland Dell and Russet Burbank, and in total free amino acids for the same varieties as well as King Edward and Maris Piper. This meant that there were significant increases in total free amino acids in all of the French fry varieties. The French fry varieties also generally contained higher concentrations of glucose and fructose than the crisping type (Fig. 2A and Fig. 2B), and showed a trend for an increase in these reducing sugars during storage and for a decline in sucrose concentration (Fig. 2C), probably as a result of invertase activity, which is often associated with potatoes in storage (cold sweetening). There were large increases in reducing sugars in King Edward, Pentland Dell, Russett Burbank and Umatilla Russett. In contrast, Daisy, Markies and Maris Piper showed little change in concentration of either reducing sugar even though sucrose concentration fell. These varieties also showed good stability during storage in a previous study (Halford *et al.*, 2012*b*).

Sucrose concentrations in the crisping varieties also fell (Fig. 2C), with the exception of Lady Rosetta, in which sucrose concentration actually rose. The concentrations of reducing sugars in Lady Rosetta also rose, suggesting that sucrose was being broken down but that the replenishing of sucrose concentrations through the breakdown of starch was occurring more rapidly in this variety. Both Lady Claire and Verdi showed a decrease in reducing sugar concentration during storage, resulting in them having the lowest reducing sugar concentrations of all the varieties at the end of the storage period. In contrast, the sucrose concentration in the boil type, Harmony was very low, while both fructose and glucose concentrations were extremely high and rose even higher through storage, suggesting high invertase activity.

**Figure 3:**
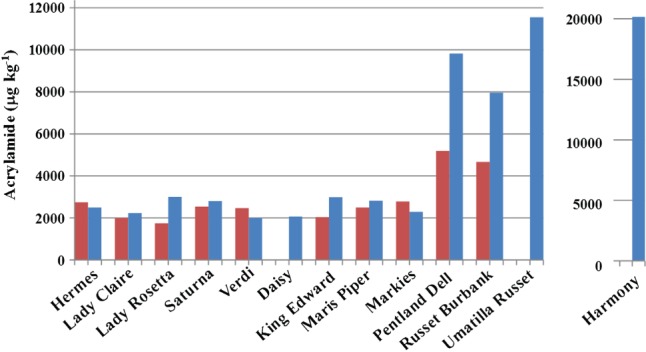
Acrylamide formation (µg kg^−1^ dry weight) in tuber flour heated to 160°C for 20 minutes for 13 potato varieties before storage (red) and after storage (blue). Harmony is shown separately on a different scale because of the relatively high concentration of acrylamide that formed in flour from that variety. Back-transformed means from analysis of variance (ANOVA) are shown except for Daisy, Umatilla Russet and Harmony, which were not included in the ANOVA because of spoilage of the unstored samples and for which the raw mean for the stored samples is shown for comparison. Statistical analysis of the data is given in Tables 1 and 3.

Acrylamide formation in heated flour was affected significantly (*P* < 0.05, *F*-test) by type, variety nested within type, storage, an interaction between type and storage (showing that the types were affected differently by storage with respect to acrylamide-forming potential), and by variety nested within type interacting with storage (showing that different varieties within each type were also affected differently by storage). The relevant means are given in Table 3 and the back-transformed means are plotted in Fig. 3. The raw means for the stored Harmony, Umatilla Russet and Daisy are also plotted for comparison. Acrylamide concentration is given in µg kg^−1^, which is equivalent to ppb and is the unit generally preferred by the food industry and regulatory authorities; 1 µg kg^−1^ represents 14 nmol kg^−1^.

**Table 3:** Variety nested within type by storage means (*n* = 12) for acrylamide (µg kg^−1^, dry weight) (ppb) on the natural log_e_ scale, following ANOVA analyses, for stored and unstored potato samples^a^

Type	Variety	Acrylamide	
		Unstored	Stored
**Crisping**	**Hermes**	7.918	7.823
	**Lady Claire**	7.601	7.713
	**Lady Rosetta**	7.465	8.007
	**Saturna**	7.841	7.937
	**Verdi**	7.809	7.603
**French fry**	**King Edward**	7.618	8.003
	**Maris Piper**	7.823	7.945
	**Markies**	7.933	7.736
	**Pentland Dell**	8.555	9.191
	**Russet Burbank**	8.446	8.982
**SED for means with same variety (29 df)**		0.1564	
**LSD (5%)**		0.3198	
**SED for other comparisons (56 df)**		0.1548	
**LSD (5%)**		0.3102	

a The standard error of the difference (SED), degrees of freedom (df) and the least significant difference (LSD) values at the 5% level of significance for comparison of means on the log scale are also included. Daisy, Umatilla Russet and Harmony were not included in the analysis because of spoilage of the unstored samples.

Acrylamide concentrations in flour from the unstored potatoes ranged from 1745 µg kg^−1^ for Lady Rosetta to 5191 µg kg^−1^ for Pentland Dell (back-transformed means), the very high levels reflecting the method used to induce acrylamide formation. The amount of acrylamide that forms in heated potato flour is a measure of acrylamide-forming potential and commercial products would be expected to exhibit proportionally lower levels because of less severe heating. Nevertheless, such levels emphasise the necessity of controlling acrylamide formation through consistency in processing, and very high levels of acrylamide have been found in a small number of commercial potato crisp samples (Powers *et al.*, 2013). There were significant (*P* < 0.05, LSD) increases after storage for crisping variety Lady Rosetta and French fry varieties King Edward, Pentland Dell and Russet Burbank, with Pentland Dell and Russet Burbank producing 9816 and 7957 µg kg^−1^ acrylamide, respectively (back-transformed means). Markies and Verdi, on the other hand, actually showed a decrease, albeit not significant (*P* > 0.05, LSD), in acrylamide formation after storage. The stored Umatilla Russett and Harmony potatoes showed the highest levels of acrylamide, with 11 550 and 20 160 µg kg^−1^, respectively.

The effect of N was not considered in this study or included in Table 1 because it has been reported on already (Muttucumaru *et al.*, 2013) but, because samples from three different N treatments were analysed, it had to be included in the ANOVA. So, for the record, there was a significant N by storage interaction (*P* < 0.05, *F*-test) for amino acids glycine, threonine, serine, asparagine, glutamine, glutamic acid, lysine and tyrosine, a main effect of N (*P* = 0.003, *F*-test) for proline, and a type by N interaction (*P* < 0.001, *F*-test) for sucrose. There were no significant (*P* < 0.05, *F*-test) interactions between type, N and storage, or between variety nested in type, N and storage for any measured variable.

### The relationship between precursor concentration and acrylamide formation

**Figure 4:**
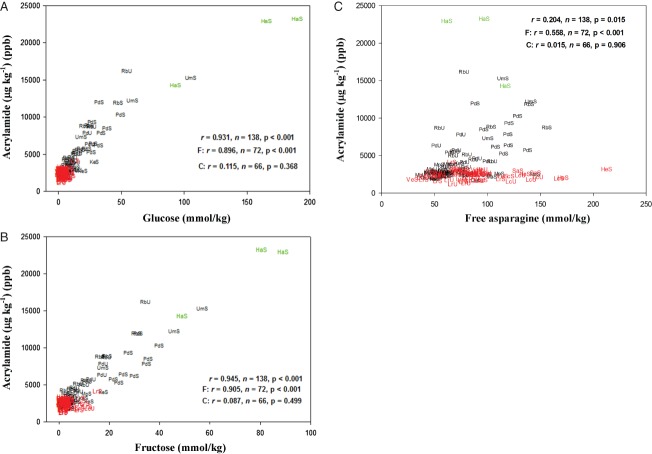
Graphs showing correlations between precursor concentration and acrylamide formation in potato flour heated to 160°C for 20 min. (A) Glucose concentration and acrylamide formation. (B) Fructose concentration and acrylamide formation. (C) Free asparagine concentration and acrylamide formation. Points on the graphs from French fry varieties are denoted by F in black, while those for crisping varieties are denoted by C in red and the boiling variety, Harmony, in green, the results for correlation (*r*) being given for all three types overall and then for French fry and crisping types separately. The points are codes for the varieties Maris Piper (Mp), Pentland Dell (Pd), King Edward (Ke), Daisy (D), Markies (Ma), Russet Burbank (Rb), Umatilla Russet (Ur), Lady Claire (Lc), Lady Rosetta (Lr), Saturna (Sa), Hermes (He), Verdi (Ve) and Harmony (Ha), followed by unstored (U) or stored (S). All concentrations are shown on a dry weight basis.

The data were analysed for correlations between precursor concentration and acrylamide formation and the results for glucose, fructose, free asparagine and acrylamide formation in flour are shown in Fig. 4A–4C. The analysis showed a strong correlation (*r* = 0.931, *P* < 0.001) between glucose concentration and acrylamide formation in the complete dataset and in the French fry varieties analysed separately (*r* = 0.896, *P* < 0.001). No significant correlation was evident in the crisping varieties (*r* = 0.115, *P* = 0.368) but this was affected by the very narrow range of glucose concentrations in these varieties, and notably the data points lie on the same relationship as those from the French fry and boil types, albeit clustered at one end of it (Fig. 4A). There was much less spread in the glucose data for the crisping varieties obtained in this study, in which all of the varieties were grown together in the same field trial, than in the data obtained from potatoes that had been grown at different sites by commercial suppliers (Halford *et al.*, 2012*b*). This suggests that these varieties are genetically very similar with respect to this trait, possibly as a result of their being bred for low glucose concentration over many years, in which case further reductions in glucose concentration may be difficult to achieve. Similar results were obtained for fructose (Fig. 4B; overall *r* = 0.945, *P* < 0.001; French fry varieties *r* = 0.905, *P* < 0.001; crisping varieties *r* = 0.087, *P* = 0.499).

The analysis also showed a significant but weak correlation between free asparagine concentration and acrylamide formation (Fig. 4C), overall (*r* = 0.204, *P* = 0.015), although more strongly in the French fry varieties (*r* = 0.558, *P* < 0.001). In contrast, there was no significant correlation in the crisping varieties (*r* = 0.015, *P* = 0.906) and, in this case, the lack of a correlation could not be explained by the range of the data points, which was greater in the crisping than in the French fry varieties.

### The relationship between glucose and fructose concentration, and between free asparagine and total free amino acids

**Figure 5:**
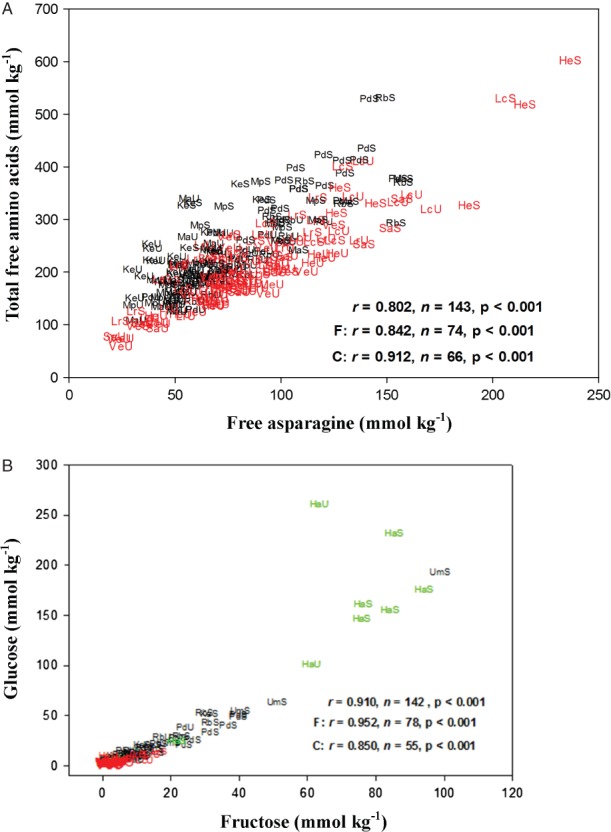
Graphs showing correlations between metabolite concentrations in potato tubers: (A) Total free amino acid and free asparagine concentration; (B). Glucose and fructose concentration. Points on the graphs from French fry varieties are denoted by F in black, while those for crisping varieties are denoted by C in red and the boiling variety, Harmony, in green, the results for correlation (*r*) being given for all three types overall and then for French fry and crisping types separately. The points are codes for the varieties Maris Piper (Mp), Pentland Dell (Pd), King Edward (Ke), Daisy (D), Markies (Ma), Russet Burbank (Rb), Umatilla Russet (Ur), Lady Claire (Lc), Lady Rosetta (Lr), Saturna (Sa), Hermes (He), Verdi (Ve) and Harmony (Ha), followed by unstored (U) or stored (S). All concentrations are shown on a dry weight basis. The total free amino acids did not include arginine, cysteine or, for the stored samples, histidine.

Previous studies have suggested that the ratio of free asparagine to total free amino acids could be an important determinant of acrylamide-forming potential, because other free amino acids could compete with asparagine in the final stages of the Maillard reaction (Elmore *et al.*, 2007; Parker *et al.*, 2012). This would be consistent with the significant reduction in acrylamide formation that has been achieved by adding amino acids to potato or cereal products, or to model systems, before heating (Bråthen *et al.*, 2005; Becalski *et al.*, 2003; Claeys *et al.*, 2005; Cook & Taylor, 2005; Low *et al.*, 2006, Rydberg *et al.*, 2008), and manipulating this parameter could have the benefit of reducing acrylamide formation while having relatively little effect on the production of desirable colour, flavour and aroma compounds that also derive from the Maillard reaction. In order to assess the variation in this trait between the different varieties, free asparagine concentration was plotted against total free amino acid concentration (Fig. 5A). This showed a close correlation between the two (*r* = 0.802, *P* < 0.001), meaning that there was little variation in the ratio of free asparagine to total free amino acids and it was not possible to assess the importance of this parameter from this dataset.

Another ratio that was suggested as potentially important by Parker *et al.* (2012) was that of fructose to glucose, the authors suggesting that a high fructose: glucose ratio could favour desirable products of the Maillard reaction (such as colour) over acrylamide. However, a plot of fructose against glucose showed that the concentrations of these two reducing sugars were also closely related (*r* = 0.91, *P* < 0.001).

### Modelling the acrylamide data

**Figure 6:**
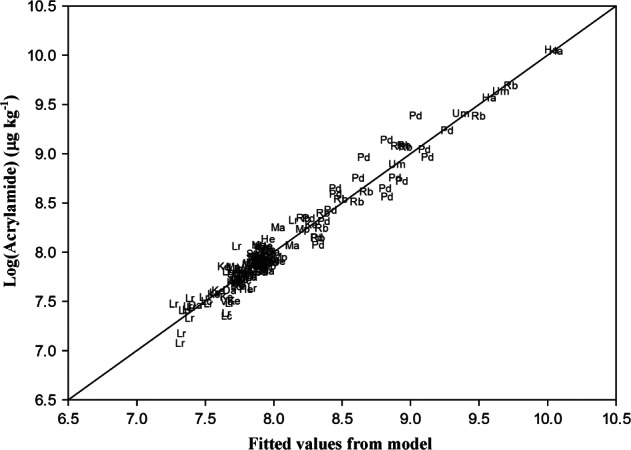
Observed acrylamide levels (µg kg^−1^ on the log_e_ scale) plotted against levels predicted by the regression model Log(Acrylamide) = Constant + *b*_1_*Fructose + *b*_2_*Sucrose + *b*_3_*Asn + *b*_4_*Pro + *b*_5_*Glucose + *b*_6_*Tyr + Variety*_i_* + Fructose·Variety*_i_* for 13 varieties of potato: Maris Piper (Mp), Pentland Dell (Pd), King Edward (Ke), Daisy (D), Markies (Ma), Russet Burbank (Rb), Umatilla Russet (Ur), Lady Claire (Lc), Lady Rosetta (Lr), Saturna (Sa), Hermes (He), Verdi (Ve) and Harmony (Ha). The line indicates the 1:1 relationship (*R*^2^ = 94.2%, *s*^2^ = 0.0195 on 107 df).

A model for the acrylamide data was derived using regression analysis with stepwise forward selection of most significant terms. This involves testing the addition of each variable to the model and adding the variable that improves the model the most. The process is repeated until no further variables improve the model. The best model was:

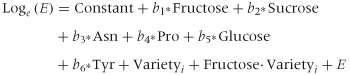
for *i* = 1…13 varieties, *b_j_* (*j* = 1…6) regression coefficients, *E* error term, and where the dot indicates the interaction (between fructose and variety). Estimates and standard errors of the parameters are given in Table 4. The fitted values from the model are plotted against the observed values in Fig. 6, with the 1:1 line indicating the quality of the fit. Fructose concentration explained most (72.07%) of the variance, with glucose concentration and sucrose concentration contributing 1.55% and 1.52%, respectively. Note that the relatively low percentage assigned to glucose compared with fructose reflects the stepwise forward selection method that was used and the similar relationship between either of these two sugars and acrylamide (Fig. 4). The fact that sucrose concentration was also significant is interesting in the light of the report that sucrose can participate in the Maillard reaction if it first undergoes enzymatic, thermal or acid-catalysed hydrolysis (De Vleeschouwer *et al.*, 2009).

**Table 4:** Parameter estimates and standard errors for multiple regression model for acrylamide data (µg kg^−1^ on the log scale): Log_e_(Acrylamide) = Constant + *b*_1_^*^Fructose + *b*_2_^*^Sucrose + *b*_3_^*^Asn + *b*_4_^*^Pro + *b*_5_^*^Glucose + *b*_6_^*^Tyr + Variety*_i_* + Fructose·Variety*_i_* for 13 varieties of potato (*R*^2^ = 94.2%, *s*^2^ = 0.0195 on 107 df)

Parameter	Estimate (SE)
Constant	5.27 (1.42)
*b*_1_	0.5495 (0.3351)
*b*_2_	0.007438 (0.002370)
*b*_3_	0.001709 (0.000727)
*b*_4_	−0.00706 (0.00272)
*b*_5_	0.001586 (0.000270)
*b*_6_	−0.1143 (0.0726)
Variety*_i_* Effects	Estimate (SE)	Fructose·Variety*_i_* Effects
**Crisping**		
Hermes	2.47 (1.42)	−0.5892 (0.3423)
Lady Claire	2.30 (1.42)	−0.5712 (0.3351)
Lady Rosetta	1.80 (1.42)	−0.4955 (0.3351)
Saturna	2.31 (1.43)	−0.5081 (0.3405)
Verdi	2.32 (1.43)	−0.5514 (0.3423)
**French Fry**		
Daisy	0 (^*^, reference)	0 (^*^, reference)
King Edward	2.14 (1.42)	−0.5027 (0.3351)
Maris Piper	2.04 (1.42)	−0.4739 (0.3351)
Markies	2.21 (1.42)	−0.4703 (0.3351)
Pentland Dell	2.64 (1.42)	−0.5207 (0.3351)
Russet Burbank	2.67 (1.42)	−0.5081 (0.3351)
Umatilla Russet	3.16 (1.44)	−0.5351 (0.3351)
**Boil**		
Harmony	3.40 (1.47)	−0.5369 (0.3351)

Asparagine also contributed to acrylamide variance (1.2%), as did two other amino acids, proline (1.1%) and tyrosine (0.6%), and there was an additional effect of variety (14.7%) that was not explained by amino acids or sugars plus a variety by fructose interaction (2.7%). These last effects indicate that a genetic component is involved, and that even within-variety changes in levels of sugars (fructose here) can be important. There is no current explanation for the contribution of tyrosine to the variance from what is known about the chemistry of acrylamide formation. Proline has been shown to inhibit acrylamide formation rather than increase it (Koutsidis *et al.*, 2009) but that would be unlikely to occur when proline is present at much lower concentrations than asparagine, as is the case here. Importantly, the effects of storage and N were not significant (*P* < 0.05, *F*-tests) in the model so there are no fundamental shifts in acrylamide with respect to such factors.

A similar analysis has been performed with data on acrylamide formation in heated rye flour (Postles *et al.*, 2013). The model arrived at in that case was:

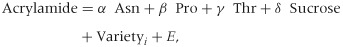
for *i* = 1…5 varieties and with estimates and standard errors of parameters (*R*^2^ = 75.6%, *s*^2^ = 11 206 on 76 df): *α* = 39.7 (13.8), *β* = 156.8 (29.1), *γ* = 536 (209), *δ* = 20.4 (18.6), variety Agronom 751.2 (90.5), Askari 824.7 (99.1), Festus 877.0 (100.0), Fugato 839.2 (99.9) and Rotari 887.0 (104.0). In that case, asparagine concentration explained most of the variance in acrylamide formation (61.8%). Proline also contributed to the variance but in a positive manner, rather than in the negative manner shown in the potato model (Table 4), with threonine also having a small effect. As with the potato model, sucrose was found to contribute marginally and there was also an effect of variety.

Regression analysis is a purely statistical exercise, but the contrasting models do reflect the fact that free asparagine concentration is the major determinant of acrylamide-forming potential in cereal flour (Curtis *et al.*, 2009, 2010; Granvogl *et al.*, 2007; Muttucumaru *et al.*, 2006; Postles *et al.*, 2013), while sugars are more important in potato, and the fact that other amino acids than asparagine contribute to the variance in both models suggests that this is worthy of further investigation.

## Discussion

Establishing the relationship between acrylamide precursor concentration in potatoes and the formation of acrylamide during cooking and processing is extremely important to enable food producers to achieve optimal quality control. Breeders are also more likely to invest in programmes aimed at reducing acrylamide-forming potential in potato if they are confident that the correct target traits have been identified. However, despite a decade of study, it has remained an intractable problem, with different studies showing reducing sugar concentration, free asparagine concentration or free asparagine concentration as a proportion of the total free amino acid pool to be the determining factor (Amrein *et al.*, 2003; Becalski *et al.*, 2004; Elmore *et al.*, 2007, 2010). This is in contrast to the situation in wheat and rye, for example, where free asparagine concentration is clearly the key parameter (Curtis *et al.*, 2009, 2010; Granvogl *et al.*, 2007; Muttucumaru *et al.*, 2006; Postles *et al.*, 2013).

Recently we reported that glucose and fructose showed the best correlations with acrylamide formation in both crisps and heated flour produced from nine varieties of potatoes grown commercially in the UK in 2009 (Halford *et al.*, *b*^2012^). However, free asparagine and total free amino acid concentrations also correlated with acrylamide formation in French fry varieties. In this study we analysed a larger number of varieties in a controlled field trial. As in the previous study, while glucose and fructose concentrations showed the best correlations with acrylamide formation, free asparagine was shown to contribute to the variance in the French fry varieties. This means that two studies have now shown free asparagine concentration to be an important contributor to acrylamide-forming potential in French fry varieties but not crisping varieties.

Another recent study modelled the kinetics of acrylamide formation in French fry production and concluded that both the fructose/glucose ratio and the ratio of asparagine to total free amino acids could affect acrylamide formation (Parker *et al.*, 2012). That study used potatoes of variety Ranger Russet, a popular French fry variety in the USA that was not included in this study. However, our finding that free asparagine concentration correlated with acrylamide formation in the French fry varieties is consistent with the predictions of the kinetic model. Total free amino acid concentrations also correlated positively with acrylamide formation in this study for French fry varieties (*r* = 0.558, *P* < 0.001, *n* = 72), which might appear to be at odds with the model. However, total free amino acid concentrations also correlated closely with free asparagine concentration, and a different picture could emerge if total free amino acid concentration could be uncoupled from free asparagine concentration. Similarly, glucose and fructose concentrations correlated closely in all of the samples, so the dataset did not test that aspect of the kinetic model either. Clearly, there are many varieties and genotypes of potato beyond those that were analysed here, but the data suggest that naturally occurring genotypes in which these relationships between precursors are different from the norm may be rare.

Very low free asparagine concentration has been achieved in genetically modified (GM) potatoes in which asparagine synthetase gene expression in the tubers has been reduced by RNA interference (Rommens *et al.*, 2008; Chawla *et al.*, 2012). These potatoes were reported to give good colour when fried, supporting the hypothesis that targeting free asparagine concentrations could enable acrylamide-forming potential to be reduced without compromising the characteristics that consumers demand in fried and roasted potato products. The strategy of targeting asparagine synthesis specifically in the tuber makes sense because asparagine has been shown not to be a major transported amino acid in potato, so the free asparagine that accumulates in tubers must be synthesised there (Muttucumaru *et al.*, 2014). Potatoes with low free asparagine rather than reducing sugar concentration could be suitable for home cooking, where most consumers use colour development to assess when roasted or fried potatoes have been cooked sufficiently. The implications of the acrylamide issue for home cooking have received very little attention so far.

Currently there is no market for GM potatoes even in the USA and there will be a lot of interest in the attitude of the all-important American fast-food industry and consumers to low acrylamide GM potatoes if and when such varieties are commercialised. There is, of course, no prospect of a GM variety being developed for the European market in the foreseeable future. However, there are steps that food manufacturers can take in conjunction with growers to ensure that they have the best possible raw material to keep acrylamide formation in their products as low as reasonably achievable. The first is variety selection; in this study the acrylamide formed in heated flour in the crisping varieties before storage ranged from 1745 (Lady Rosetta) to 2746 µg kg^−1^ (Hermes), a difference of 36%, and in the French fry varieties it ranged from 2034 (King Edward) to 5191 µg kg^−1^ (Pentland Dell). Clearly it is essential to select the right type of potato and, if possible, the best variety within the type, although other factors will also have to be considered, from the variety’s suitability for a particular environment to the quality of the product that can be made with it.

The second consideration is management of the crop, with the most important management factor identified to date being storage. In this and a previous study (Halford *et al.*, *b*^2012^), storage had a significant, variety-dependent impact on acrylamide-forming potential, with sugar and amino acid concentrations changing markedly, and an increase in reducing sugar concentrations driving up acrylamide-forming potential in most varieties. The results emphasised the importance of not using potatoes to make foods in which acrylamide may form if they have been kept beyond their recommended storage window. The varieties that were most stable during the 6-month storage period used in this study were Lady Claire and Verdi from the crisping types and Markies and Maris Piper from the French fry types. Two of the varieties in the trial, Lady Rosetta (crisping) and Pentland Dell (French fry), are regarded as having particularly rigid storage windows, with both having a reputation for exhibiting senescent sweetening relatively early in storage. Both of these varieties showed large increases in reducing sugar concentration after storage in this study, emphasising the importance of processing them after short- to medium-term storage and not holding them long-term. King Edward, Russet Burbank, Umatilla Russet (all French fry type) and Harmony (boiling) also showed large increases in reducing sugars after storage.

The food industry has worked hard to reduce the levels of acrylamide in its products and there has been impressive progress in some sectors, with levels of acrylamide in potato crisps in Europe, for example, falling by 53% between 2002, when acrylamide was first discovered in food, and 2011 (Powers *et al.*, 2013). There is certainly an expectation within the food industry that plant breeders will engage with the issue with the same determination, and some frustration that there has not been more progress. It is important that potato breeders make reduced acrylamide-forming potential a priority and that they have sufficient information on the genetics underlying what is undoubtedly a complex trait. The important conclusion from this study is that free asparagine as well as reducing sugar concentration should be a target. Indeed, we concur with the conclusion of Parker *et al.* (2012) that reducing the concentration of free asparagine as a proportion of the total free amino acid pool would be the most likely way of reducing acrylamide formation in potato products while retaining the characteristics that define products and are demanded by consumers, bearing in mind again that compounds responsible for colour, flavour and aroma form by similar pathways to acrylamide.

## Supporting Information

Appendix S1 The complete data set on free amino acid, glucose, fructose and sucrose concentrations in the potato samples, and on the levels of acrylamide formed in heated flour.
